# The difficult case of an RNA-only origin of life

**DOI:** 10.1042/ETLS20190024

**Published:** 2019-08-28

**Authors:** Kristian Le Vay, Hannes Mutschler

**Affiliations:** Max-Planck Institute of Biochemistry, Am Klopferspitz 18, 82152 Martinsried, Germany

**Keywords:** origins of life, prebiotic chemistry, RNA catalysis, RNA world, self-replication

## Abstract

The RNA world hypothesis is probably the most extensively studied model for the emergence of life on Earth. Despite a large body of evidence supporting the idea that RNA is capable of kick-starting autocatalytic self-replication and thus initiating the emergence of life, seemingly insurmountable weaknesses in the theory have also been highlighted. These problems could be overcome by novel experimental approaches, including out-of-equilibrium environments, and the exploration of an early co-evolution of RNA and other key biomolecules such as peptides and DNA, which might be necessary to mitigate the shortcomings of RNA-only systems.

The conjecture that life on Earth evolved from an ‘RNA World’ remains one of the most popular hypotheses for abiogenesis, even 60 years after Alex Rich first put the idea forward [1]. For some, evidence based upon ubiquitous molecular fossils and the elegance of the idea that RNA once had a dual role as information carrier and prebiotic catalyst provide overwhelming support for the theory. Nevertheless, doubts remain surrounding the chemical evolution of an RNA world, whose classical scenario is based on a temporal sequence of nucleotide formation, enzyme-free polymerisation/replication, recombination, encapsulation in lipid vesicles (or other compartments), evolution of ribozymes and finally the innovation of the genetic code and its translation (Figure 1) [2,3]. Common criticisms are that RNA is too complex to emerge *de novo* in a prebiotic environment, that catalysis is a relatively rare property of RNA and requires implausibly long strands, that the catalytic repertoire of RNA is too limited and that it is difficult to envisage scenarios in which precursors and feedstocks occurred at sufficient concentrations to allow replication and evolution [4].

**Figure 1.**
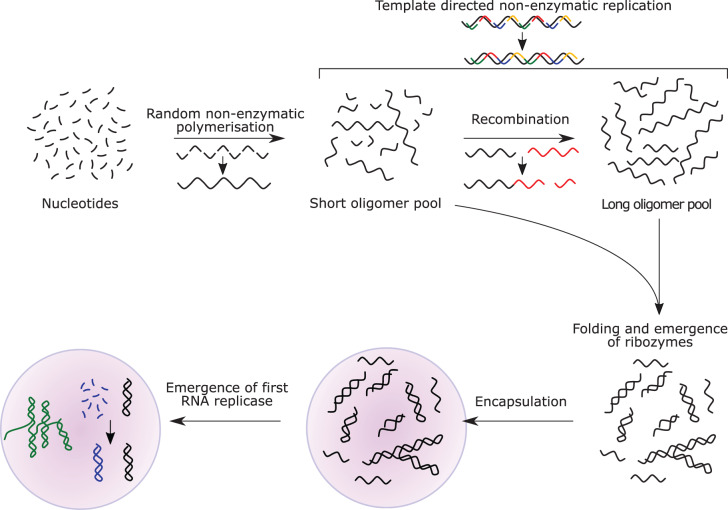
A schematic representation of the classical RNA world hypothesis. Initially, synthesis and random polymerisation of nucleotides result in pools of nucleic acid oligomers, in which template-directed non-enzymatic replication may occur. Recombination reactions result in the generation of longer oligomers. Both long and short oligomers can fold into structures of varying complexity, resulting in the emergence of functional ribozymes. As complexity increases, the first RNA replicase emerges, and encapsulation results in protocells with distinct genetic identities capable of evolution. In reality, it is likely that multiple processes occurred in parallel, rather than in a strictly stepwise manner, and encapsulation may have occurred at any stage.

Breakthroughs in prebiotic chemistry demonstrating how the essential building blocks of RNA (and other biomolecules) may have formed under different primordial scenarios address many concerns about the plausibility of RNA or related nucleic acid emergence in a prebiotic world [5–9]. Similarly, demonstrations of enzyme-free polymerisation and copying of nucleic acids from activated building blocks [10–13] and the innate potential of random RNA strands to recombine and ligate show that the emergence of longer RNA strands capable of catalysis is, in principle, feasible [14–16]. The *in vitro* selection of ribozymes has over the years revealed the impressive catalytic repertoire of nucleic acids, despite their conformal and sequence-based limitations compared with proteins [17]. RNA is particularly adept at manipulations of its own phosphate backbone — precisely the chemistry needed to catalyse self-replication.

Despite these rebuttals, it has not yet been possible to demonstrate robust and continuous RNA self-replication from a realistic feedstock (i.e. activated mono- or short mixed-sequence oligonucleotides). Major obstacles for RNA copying such as efficiency, regiospecificity and fidelity and are discussed elsewhere [18,19] but are mostly true for both non-enzymatic and enzymatic scenarios. The ever-looming strand dissociation problem is of particular concern (Figure 2). The high melting temperature (*T*_m_) of long RNA duplexes, such as those that arise from template-directed replication, results in the formation of dead-end duplex complexes in the absence of highly evolved helicases. When complementary RNA strands are separated, for example, by heat denaturation, reannealing occurs orders of magnitudes faster than known copying reactions [18].

**Figure 2.**
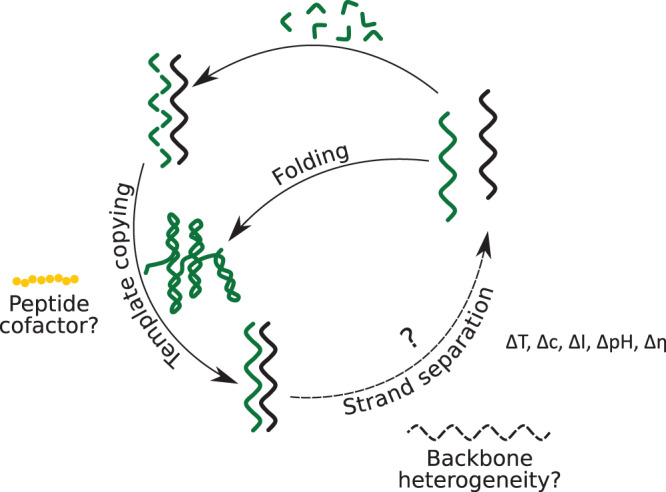
A schematic illustrating nucleic acid replication and the strand separation problem. Template copying, both enzymatic and non-enzymatic, is well established. However, the melting temperature (*T*_m_) of the resulting nucleic acid duplex is typically high, and as such, strand dissociation unfavourable and replication cycles are strongly inhibited. Possible solutions include lowering duplex *T*_m_ by introducing backbone heterogeneity or non-equilibrium conditions such as fluctuations in temperature (*T*), concentration (*c*), ionic strength/divalent ion concentration (*I*), pH or viscosity (*η*).

In the case of ribozymes, only ‘simple’ ligation or recombination-based RNA replication from defined oligonucleotides has been demonstrated [20–23]. Such systems have only a limited ability to transmit heritable information and so are not capable of open-ended evolution — the ability to indefinitely increase in complexity like living systems [24]. Open-ended evolution requires that a replicase must at least be able to efficiently copy generic sequences longer than that required to encode its own function. This topic has been reviewed in detail elsewhere [24,25].

The search for an RNA replicase ribozyme, a cornerstone of the RNA world hypothesis, is largely founded on improvements of the scaffold of the R18 RNA polymerase ribozyme, which itself is an optimised version of the complex class I ligase ribozyme [26]. The discovery of the class 1 ligase, which is capable of ligating RNA with higher efficiency and better turnover than most ribozymes, was perhaps a lucky coincidence (or misfortune, if better ribozymes were missed), and can optimistically be expected to occur on average every 1 in 2000 selection experiments [27]. Considering this, it is truly astonishing how far this single ribozyme family has been developed. Initially capable of copying only very simple templates [27], variants of the polymerase are now able to copy complex templates [28,29], including the synthesis of an entire catalytic domain of a polymerase itself from trinucleotides [30], achieved through copying and subsequent ligation of fragments albeit with multiple human interventions. The much anticipated ‘riboPCR’, the amplification of RNA sequences in a ribozyme-catalysed polymerase chain-like reaction, has so far only been successful for very short primer dimers, which can be melted rapidly at relatively low temperatures, therefore minimising temperature-induced RNA hydrolysis [29]. The apparent limitations of riboPCR with respect to amplification of long strands complicate self-replication scenarios, although schemes evoking the asymmetric replication of short RNAs (where ‘antisense’ strands are produced in excess over coding ‘sense’ strands) followed by ligation or recombination into an active replicase could still provide an elegant solution to the problem [31,32].

Looking back at the long history of the field, one might wonder why we have yet to achieve (self-sustained) RNA replication and transcription, despite its centrality to the RNA world hypothesis. There are three possibilities:

1. RNA is capable of this process but more time is needed to identify either conditions or replicators of sufficient complexity that are able to solve the various problems associated with protein-free RNA replication.
2. RNA in isolation (including ribozymes) is simply not sufficient to catalyse its own replication, and substantial help from either other molecules or the environment is essential.
3. RNA replication was never really central during early molecular evolution but rather the late result of a (crudely) replicating non-enzymatic metabolism [33–37] or an early ‘polypeptide first’ world [38–40]. We will not discuss the merits of these scenarios here, but believe it is crucial to test and challenge the predictions made by these alternative models experimentally.

For the first possibility, it may only be a matter of time and combined efforts to identify experimental model scenarios that are convincing enough to please critics of the field. In the worst case, the formation of life as we know it from RNA could be the result of a ‘frozen accident’, similar to the genetic code [41], that is generally hard or impossible to reproduce (e.g. a robust self-replicating ribozyme). However, the current consensus seems to be that while it may never be possible to identify the exact trajectory that led to our modern biochemistry, it should still be possible to emulate the process and find related routes that lead to a ‘recapitulated’ origin *ex situ* [42]. This notion is largely grounded on the assumption that enzyme-free replication requires no *a priori* sequence information and can, therefore, emerge spontaneously under suitable environmental and chemical conditions (e.g. a continuous supply of activated monomers and processes that enable repeated strand separation). Assuming the remaining experimental problems of continuous enzyme-free nucleic acid replication are solved, natural selection should spontaneously produce systems that are better or ‘good enough’ to persist under the given conditions. Whether these new replicators will necessarily evolve into more complex systems with advanced (and potentially emergent) properties (e.g. cooperative ribozyme networks) is of course another matter, although simulations predict that genomic complexity is forced to increase in a fixed environment [43–45].

To identify suitable conditions for such an in-laboratory origin, a ‘flexible’ approach is probably the best choice in light of the large number of possible geochemical conditions that have been proposed to host the emergence of life [46]. In other words, it is most sensible to perform key experiments under relaxed but plausible experimental boundary conditions instead of trying to implement strict restraints based on educated guesses about a specific prebiotic environment. Once a set of experimental conditions that can sustain certain crucial reactions such as RNA synthesis, building block activation and self-replication have been identified, it will help to pinpoint plausible geochemical scenarios automatically. There are several examples of such problem-oriented approaches, e.g. tackling the strand inhibition problem during the replication of long RNAs using viscous solvents and temperature oscillations [47], overcoming low substrate concentrations and the fragility of RNA by working under frozen conditions [48,49] or implementation of scenarios enabling multistep, uninterrupted synthesis of key building blocks of nucleotide synthesis [50] or nucleotides themselves [16]. In addition, combining typical model RNA world reactions with non-equilibrium settings based upon thermal gradients shows great promise [51]. For example, gas bubbles in combination with thermal gradients cause dissolved materials to cycle between dry and wet states and enable the key steps of precursor/oligonucleotide accumulation and RNA phosphorylation, while drastically increasing ribozyme activity and facilitating RNA encapsulation into vesicle aggregates [52]. Oscillating salt concentrations in such environments cause local melting of nucleic acid duplexes up to 20°C lower than the *T*_m_, which could provide an environmental route to overcoming the strand dissociation problem [53]. It remains to be seen if such environments can eventually support coupled cycles of RNA activation, replication and encapsulation under continuous conditions.

Similar combined efforts will also be necessary if RNA alone is insufficient to drive continuous self-replication and evolution. In this case, it may be necessary to diversify the pool of feedstock molecules by taking into account the chemical and conformational heterogeneities found in many experimental scenarios. For example, nucleic acid polymers with non-inheritable backbone heterogeneities (e.g. 2′–5′ versus 3′–5′ backbone heterogeneity for RNA [54] or mixed nucleotide backbones formed from RNA, DNA or other nucleic acid types [55]) have fascinating properties. In particular, some chimeric backbones decrease duplex stabilities, which could help to mitigate the strand dissociation problem [56]. Such a ‘mixed’ scenario seems plausible in view of the prebiotic clutter [57]. Recent synthesis strategies coming from different laboratories have found strong evidence that RNA and DNA could have arisen from the same set of precursor molecules [9,58], and ribozymes that can read and write both nucleotide backbone chemistries have already been found [59]. Even though heterogeneous nucleic acids pose a general problem for hereditability of genetic information, such chimaeras could have played an important role as non-genetic catalysts similar to modern proteins. Exploring such heterogeneous scenarios poses major experimental challenges, as many of the standard tools used to study RNA, particularly reverse transcription and (deep) sequencing, are harder or impossible with mixed backbone chemistries. Nevertheless, it remains important to investigate these scenarios and, if necessary, develop new molecular biological tools that can cope with non-homogeneous nucleic acid backbones [60].

Plausible help for RNA might also come from primitive polypeptides (thoroughly discussed elsewhere [61]) Nearly, all ribozymes found in extant biology are associated with proteins that help them to carry out their function under intracellular conditions. These ribonucleoprotein complexes are thought to be remnants of an ancient biology where polypeptides could have supported folding and substrate binding of catalytic RNAs [62,63]. Before the advent of translation, these peptide cofactors would have been very simple or even comprised of a pool of random peptides with sequence biases [64]. As such, they would probably not have been initially capable of precise functionalities requiring a well-defined active site (although there might have been some notable exceptions [65]). Even such simple peptides could have been crucial for RNA protection during non-enzymatic replication [18] and during ribozyme-catalysed RNA copying [66]. It is tempting to speculate that peptides might have also granted ancient ‘ribonucleopeptide RNA replicases’ improved non-specific affinity to their substrates (i.e. a primer-template duplex), which is required for processivity but difficult to achieve with the polyanionic phosphate backbone of RNA alone. An advantage of this hypothesis is that an early co-evolution of RNA and peptides makes the transition to protein-dominated biology seem more plausible. Moreover, an early cooperation between RNA and peptides might also provide an elegant route to the formation of the first protocells before the advent of membrane-bound compartments [67]. As with nucleic acid heterogeneity, the inclusion of peptides represents an enormous analytical and experimental challenge, which will only be addressed by close collaboration between multiple disciplines within the origin of life field.

There remains the hope for origin of life scenarios where RNA plays a major role as an information carrier and catalyst. New experimental approaches using out-of-equilibrium settings could finally result in genuine RNA-based self-replicating systems capable of open-ended evolution. More complex scenarios involving RNA, DNA, peptides, simpler polynucleotides, chimeric intermediates or other yet unknown helper molecules may also be required, which will complicate the analytical understanding of the model systems and may ultimately render the term ‘RNA world’ in its traditional sense obsolete.

## Summary

- Despite advances in prebiotic chemistry, it has not yet been possible to demonstrate robust and continuous RNA self-replication from a realistic feedstock.
- RNA in isolation may not be sufficient to catalyse its own replication and may require help from either other molecules or the environment.
- Non-equilibrium environments, backbone heterogeneity and polypeptide cofactors may address some of the remaining problems in the RNA world hypothesis.

## References

[1] Rich A. (1962) Horizons in Biochemistry, Academic Press, New York, United States

[2] Pressman A., Blanco C. and Chen I.A. (2015) The RNA world as a model system to study the origin of life. Curr. Biol. 25, R953–R963 10.1016/j.cub.2015.06.016 26439358

[3] Joyce G.F. and Szostak J.W. (2018) Protocells and RNA self-replication. Cold Spring Harb. Perspect. Biol. 10, a034801 10.1101/cshperspect.a034801 30181195 PMC6120706

[4] Bernhardt H.S. (2012) The RNA world hypothesis: the worst theory of the early evolution of life (except for all the others). Biol. Dir. 7, 23 10.1186/1745-6150-7-23 PMC3495036 22793875

[5] Powner M.W., Gerland B. and Sutherland J.D. (2009) Synthesis of activated pyrimidine ribonucleotides in prebiotically plausible conditions. Nature 459, 239–242 10.1038/nature08013 19444213

[6] Patel B.H., Percivalle C., Ritson D.J., Duffy C.D. and Sutherland J.D. (2015) Common origins of RNA, protein and lipid precursors in a cyanosulfidic protometabolism. Nat. Chem. 7, 301–307 10.1038/nchem.2202 25803468 PMC4568310

[7] Becker S., Thoma I., Deutsch A., Gehrke T., Mayer P., Zipse H. et al. (2016) A high-yielding, strictly regioselective prebiotic purine nucleoside formation pathway. Science 352, 833–836 10.1126/science.aad2808 27174989

[8] Kim H.-J. and Benner S.A. (2017) Prebiotic stereoselective synthesis of purine and noncanonical pyrimidine nucleotide from nucleobases and phosphorylated carbohydrates. Proc. Natl Acad. Sci. U.S.A. 114, 11315–11320 10.1073/pnas.1710778114 29073050 PMC5664531

[9] Teichert J.S., Kruse F.M. and Trapp O. (2019) Direct prebiotic pathway to DNA nucleosides. Angew. Chem. Int. Ed. 58, 9944–9947 10.1002/anie.201903400 31131499

[10] Monnard P.A., Kanavarioti A. and Deamer D.W. (2003) Eutectic phase polymerization of activated ribonucleotide mixtures yields quasi-Equimolar incorporation of purine and pyrimidine nucleobases. J. Am. Chem. Soc. 125, 13734–13740 10.1021/ja036465h 14599212

[11] Deck C., Jauker M. and Richert C. (2011) Efficient enzyme-free copying of all four nucleobases templated by immobilized RNA. Nat. Chem. 3, 603–608 10.1038/nchem.1086 21778979

[12] O'Flaherty D.K., Kamat N.P., Mirza F.N., Li L., Prywes N., Szostak J. W. et al. (2018) Copying of mixed-Sequence RNA templates inside model protocells. J. Am. Chem. Soc. 140, 5171–5178 10.1021/jacs.8b00639 29608310 PMC7547884

[13] Prywes N., Blain J.C., Del Frate F. and Szostak J.W. (2016) Nonenzymatic copying of RNA templates containing all four letters is catalyzed by activated oligonucleotides. Elife 5, e17756 10.7554/eLife.17756 27351102 PMC4959843

[14] Lutay A.V., Zenkova M.A. and Vlassov V.V. (2007) Nonenzymatic recombination of RNA: possible mechanism for the formation of novel sequences. Chem. Biodivers. 4, 762–767 10.1002/cbdv.200790062 17443887

[15] Smail B.A., Clifton B.E., Mizuuchi R. and Lehman N. (2019) Spontaneous advent of genetic diversity in RNA populations through multiple recombination mechanisms. RNA 25, 453–464 10.1261/rna.068908.118 30670484 PMC6426292

[16] Mutschler H., Taylor A.I., Porebski B.T., Lightowlers A., Houlihan G., Abramov M. et al. (2018) Random-sequence genetic oligomer pools display an innate potential for ligation and recombination. Elife 7, e43022 10.7554/eLife.43022 PMC6289569 30461419

[17] Wachowius F., Attwater J. and Holliger P. (2017) Nucleic acids: function and potential for abiogenesis. Q. Rev. Biophys. 50, e4 10.1017/S0033583517000038 29233216

[18] Szostak J.W. (2012) The eightfold path to non-enzymatic RNA replication. J. Syst. Chem. 3, 2 10.1186/1759-2208-3-2

[19] Sosson M. and Richert C. (2018) Enzyme-free genetic copying of DNA and RNA sequences. Beilstein J. Org. Chem. 14, 603–617 10.3762/bjoc.14.47 29623122 PMC5870163

[20] Gwiazda S., Salomon K., Appel B. and Müller S. (2012) RNA self-ligation: from oligonucleotides to full length ribozymes. Biochimie 94, 1457–1463 10.1016/j.biochi.2012.03.015 22465106

[21] Robertson M.P. and Joyce G.F. (2014) Highly efficient self-replicating RNA enzymes. Chem. Biol. 21, 238–245 10.1016/j.chembiol.2013.12.004 24388759 PMC3943892

[22] Lincoln T.A. and Joyce G.F. (2009) Self-sustained replication of an RNA enzyme. Science 323, 1229–1232 10.1126/science.1167856 19131595 PMC2652413

[23] Hayden E.J. and Lehman N. (2006) Self-assembly of a group I intron from inactive oligonucleotide fragments. Chem. Biol. 13, 909–918 10.1016/j.chembiol.2006.06.014 16931340

[24] Duim H. and Otto S. (2017) Towards open-ended evolution in self-replicating molecular systems. Beilstein J. Org. Chem. 13, 1189–1203 10.3762/bjoc.13.118 28694865 PMC5496545

[25] Le Vay K., Weise L.I., Libicher K., Mascarenhas J. and Mutschler H. (2019) Templated self-replication in biomimetic systems. Adv. Biosyst. 3 10.1002/adbi.201800313 32648707

[26] Johnston W.K., Unrau P.J., Lawrence M.S., Glasner M.E. and Bartel D.P. (2001) RNA-catalyzed RNA polymerization: accurate and general RNA-templated primer extension. Science 292, 1319–1325 10.1126/science.1060786 11358999

[27] Ekland E.H., Szostak J.W. and Bartel D.P. (1995) Structurally complex and highly active RNA ligases derived from random RNA sequences. Science 269, 364–370 10.1126/science.7618102 7618102

[28] Wochner A., Attwater J., Coulson A. and Holliger P. (2011) Ribozyme-catalyzed transcription of an active ribozyme. Science 332, 209–212 10.1126/science.1200752 21474753

[29] Horning D.P. and Joyce G.F. (2016) Amplification of RNA by an RNA polymerase ribozyme. Proc. Natl Acad. Sci. U.S.A. 113, 9786–9791 10.1073/pnas.1610103113 27528667 PMC5024611

[30] Attwater J., Raguram A., Morgunov A.S., Gianni E. and Holliger P. (2018) Ribozyme-catalysed RNA synthesis using triplet building blocks. Elife 7, e35255 10.7554/eLife.35255 29759114 PMC6003772

[31] Meyer A.J., Ellefson J.W. and Ellington A.D. (2012) Abiotic self-replication. Acc. Chem. Res. 45, 2097–2105 10.1021/ar200325v 22891822

[32] Mutschler H., Wochner A. and Holliger P. (2015) Freeze-thaw cycles as drivers of complex ribozyme assembly. Nat. Chem. 7, 502–508 10.1038/nchem.2251 25991529 PMC4495579

[33] Dyson F. (1999) Origins of Life, Cambridge University Press, Cambridge, United Kingdom

[34] Kauffman S.A. (1993) The Origins of Order: Self-Organization and Selection in Evolution, Oxford University Press, New York, United States

[35] Segre D., Ben-Eli D. and Lancet D. (2000) Compositional genomes: Prebiotic information transfer in mutually catalytic noncovalent assemblies. Proc. Natl Acad. Sci. U.S.A. 97, 4112–4117 10.1073/pnas.97.8.4112 10760281 PMC18166

[36] Wächtershäuser G. (1997) The origin of life and its methodological challenge. J. Theor. Biol. 187, 483–494 10.1006/jtbi.1996.0383 9299293

[37] Vasas V., Fernando C., Santos M., Kauffman S. and Szathmáry E. (2012) Evolution before genes. Biol. Dir. 7, 1 10.1186/1745-6150-7-1 PMC3284417 22221860

[38] Wills P.R. and Carter C.W. (2018) Insuperable problems of the genetic code initially emerging in an RNA world. BioSystems 164, 155–166 10.1016/j.biosystems.2017.09.006 28903058 PMC5895081

[39] Guseva E., Zuckermann R.N. and Dill K.A. (2017) Foldamer hypothesis for the growth and sequence differentiation of prebiotic polymers. Proc. Natl Acad. Sci. U.S.A. 114, E7460–E7468 10.1073/pnas.1620179114 28831002 PMC5594640

[40] Kurland C.G. (2010) The RNA dreamtime: modern cells feature proteins that might have supported a prebiotic polypeptide world but nothing indicates that RNA world ever was. BioEssays 32, 866–871 10.1002/bies.201000058 20806270

[41] Hinegardner R.T. and Engelberg J. (1963) Rationale for a universal genetic code. Science 142, 1083–1085 10.1126/science.142.3595.1083 14068231

[42] Szostak J. (2018) How did life begin? Nature 557, S13–S15 10.1038/d41586-018-05098-w 29743709

[43] Adami C., Ofria C. and Collier T.C. (2000) Evolution of biological complexity. Proc. Natl Acad. Sci. U.S.A. 97, 4463–4468 10.1073/pnas.97.9.4463 10781045 PMC18257

[44] Adami C. (2002) What is complexity? BioEssays 24, 1085–1094 10.1002/bies.10192 12447974

[45] Yaeger L., Griffith V. and Sporns O. (2011) Passive and driven trends in the evolution of complexity, arXiv, 1112.4906 http://arxiv.org/abs/1112.4906

[46] Powner M.W. and Sutherland J.D. (2011) Prebiotic chemistry: a new *modus operandi*. Phil. Trans. R. Soc. B 366, 2870–2877 10.1098/rstb.2011.0134 21930577 PMC3158916

[47] He C., Lozoya-Colinas A., Gállego I., Grover M.A. and Hud N.V. (2019) Solvent viscosity facilitates replication and ribozyme catalysis from an RNA duplex in a model prebiotic process. Nucleic Acids Res. 47, 6569–6577 10.1093/nar/gkz496 31170298 PMC6649698

[48] Attwater J., Wochner A., Pinheiro V.B., Coulson A. and Holliger P. (2010) Ice as a protocellular medium for RNA replication. Nat. Commun. 1, 1–8 10.1038/ncomms1076 20865803

[49] Vlassov A.V., Johnston B.H., Landweber L.F. and Kazakov S.A. (2004) Ligation activity of fragmented ribozymes in frozen solution: Implications for the RNA world. Nucleic Acids Res. 32, 2966–2974 10.1093/nar/gkh601 15161960 PMC419604

[50] Ritson D.J., Battilocchio C., Ley S.V. and Sutherland J.D. (2018) Mimicking the surface and prebiotic chemistry of early earth using flow chemistry. Nat. Commun. 9, 1821 10.1038/s41467-018-04147-2 29739945 PMC5940729

[51] Agerschou E.D., Mast C.B. and Braun D. (2017) Emergence of life from trapped nucleotides? Non-equilibrium behavior of oligonucleotides in thermal gradients. Synlett 28, 56–63

[52] Morasch M., Liu J., Dirscherl C.F., Ianeselli A., Kühnlein A., Le Vay K. et al. (2019) Heated gas bubbles enrich, crystallize, dry, phosphorylate, and encapsulate prebiotic molecules. Nat. Chem. 10.1038/s41557-019-0299-5 31358919

[53] Ianeselli A., Mast C.B. and Braun D. (2019) Periodic melting of oligonucleotides by oscillating salt concentrations triggered by microscale water cycles inside heated rock pores. Angew. Chem. Int. Ed. 10.1002/anie.201907909 PMC7616952 31322800

[54] Engelhart A.E., Powner M.W. and Szostak J.W. (2013) Functional RNAs exhibit tolerance for non-heritable 2′-5′ versus 3′-5′ backbone heterogeneity. Nat. Chem. 5, 390–394 10.1038/nchem.1623 23609089 PMC4088963

[55] Efthymiou T., Gavette J., Stoop M., De Riccardis F., Froeyen M., Herdewijn P. et al. (2018) Chimeric XNA: an unconventional design for orthogonal informational systems. Chem. A Eur. J. 24, 12811–12819 10.1002/chem.201802287 29901248

[56] Gavette J.V., Stoop M., Hud N.V. and Krishnamurthy R. (2016) RNA–DNA chimeras in the context of an RNA world transition to an RNA/DNA world. Angew. Chem. Int. Ed. 55, 13204–13209 10.1002/anie.201607919 27650222

[57] Krishnamurthy R. (2017) Giving rise to life: transition from prebiotic chemistry to protobiology. Acc. Chem. Res. 50, 455–459 10.1021/acs.accounts.6b00470 28945387

[58] Xu J., Green N.J., Gibard C., Krishnamurthy R. and Sutherland J.D. (2019) Prebiotic phosphorylation of 2-thiouridine provides either nucleotides or DNA building blocks via photoreduction. Nat. Chem. 11, 457–462 10.1038/s41557-019-0225-x 30936523 PMC6597365

[59] Samanta B. and Joyce G.F. (2017) A reverse transcriptase ribozyme. eLife 6, e31153 10.7554/eLife.31153 28949294 PMC5665644

[60] Taylor A.I., Houlihan G. and Holliger P. (2019) Beyond DNA and RNA: the expanding toolbox of synthetic genetics. Cold Spring Harb. Perspect. Biol. 11, a032490 10.1101/cshperspect.a032490 31160351 PMC6546049

[61] Toparlak O.D. and Mansy S.S. (2019) Progress in synthesizing protocells. Exp. Biol. Med. 244, 304–313 10.1177/1535370218816657 PMC6435886 30509137

[62] Alva V., Söding J. and Lupas A.N. (2015) A vocabulary of ancient peptides at the origin of folded proteins. Elife 4, e09410 10.7554/eLife.09410 26653858 PMC4739770

[63] Das C. and Frankel A.D. (2003) Sequence and structure space of RNA-binding peptides. Biopolymers 70, 80–85 10.1002/bip.10429 12925994

[64] Rodriguez-Garcia M., Surman A.J., Cooper G.J.T., Suárez-Marina I., Hosni Z., Lee M. P. et al. (2015) Formation of oligopeptides in high yield under simple programmable conditions. Nat. Commun. 6, 8385 10.1038/ncomms9385 26442968 PMC4633627

[65] Kim J.D., Pike D.H., Tyryshkin A.M., Swapna G.V.T., Raanan H., Montelione G. T. et al. (2018) Minimal heterochiral *de novo* designed 4Fe–4S binding peptide capable of robust electron transfer. J. Am. Chem. Soc. 140, 11210–11213 10.1021/jacs.8b07553 30141918 PMC7050467

[66] Tagami S., Attwater J. and Holliger P. (2017) Simple peptides derived from the ribosomal core potentiate RNA polymerase ribozyme function. Nat. Chem. 9, 325–332 10.1038/nchem.2739 28338682 PMC5458135

[67] Oparin A.I. (1965) The origin of life and the origin of enzymes. Adv. Enzymol. Relat. Areas. Mol. Biol. 27, 347–380 4882862 10.1002/9780470122723.ch7

